# Venous Thromboembolism: An Unusual Presentation of Pulmonary Tuberculosis

**DOI:** 10.7759/cureus.14092

**Published:** 2021-03-24

**Authors:** Nishan K Purayil, Jaseem Sirajudeen, Khaled M Al Arbi, Mohamed A Baghi, Muhammad Zahid

**Affiliations:** 1 Internal Medicine, Hamad Medical Corporation, Doha, QAT; 2 College of Medicine, Qatar University, Doha, QAT; 3 Internal Medicine, Weill Cornell Medicine-Qatar, Doha, QAT; 4 Internal Medicine, Qatar University, Doha, QAT; 5 Internal Medicine, Hamad General Hospital, Doha, QAT; 6 Medicine, Hamad Medical Corporation, Doha, QAT

**Keywords:** tuberculosis, deep vein thrombosis (dvt), pulmonary embolism (pe), virchow’s triad

## Abstract

Tuberculosis is a leading cause of death due to infectious etiology worldwide. Myriad presentations and multisystem involvement of the disease can make the diagnosis extremely challenging. Venous thromboembolism is an uncommon entity in tuberculosis. The prevalence of venous thromboembolism is reported to be 1.5-3.4%. The etiology of thrombosis could be multifactorial. All the elements of Virchow’s triad can be present in these patients. This case report is about a patient presenting with deep vein thrombosis (DVT) and pulmonary embolism (PE), who was subsequently diagnosed with active pulmonary tuberculosis.

## Introduction

Despite the global efforts to control tuberculosis (TB), the burden of this disease remains high and is still a leading cause of death due to infectious etiology [1]. TB presents with various pulmonary or extrapulmonary manifestations. Venous thromboembolism, that is deep vein thrombosis (DVT) and pulmonary embolism (PE) are uncommon in TB, with a reported prevalence of around 1.5-3.5% [2]. The occurrence of both in the same patient is rarer. Even though DVT and PE belong to the same spectrum of a disease, the former entity is three times more prevalent than the latter in patients with TB [3].

## Case presentation

**Figure 1:**
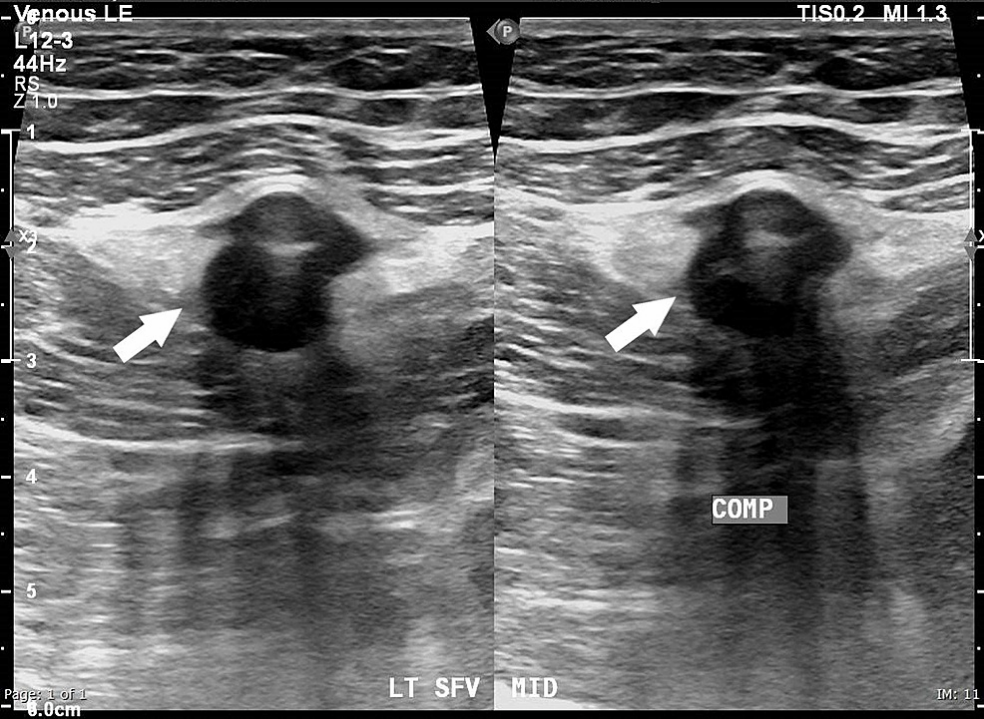
Grey-scale ultrasound at the level of the mid-thigh demonstrating non-compressibility of the superficial femoral vein (white arrows)

**Figure 2:**
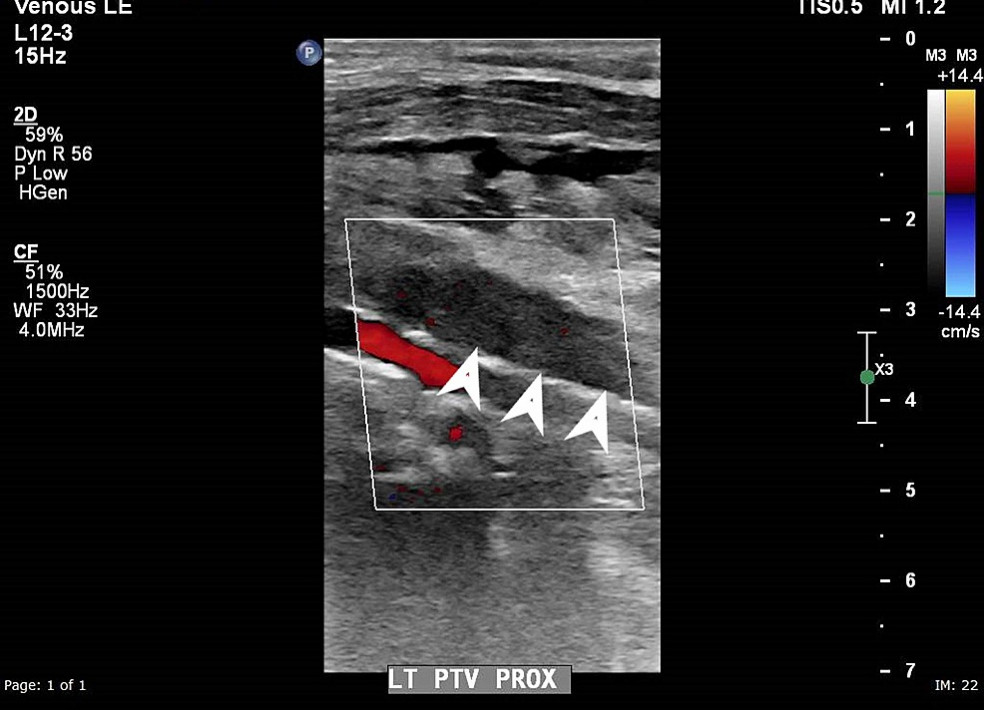
Colour Doppler ultrasound at the level of the proximal calf demonstrating absent colour flow in the posterior tibial vein (arrowheads) suggestive of thrombosis

A 49-year-old gentleman from the Asian subcontinent, who was not known to have any significant medical illnesses in the past was admitted with unilateral lower limb swelling of three days duration and was subsequently diagnosed to have DVT. He denied recent immobilization, hospitalization or travel. There was no history of trauma, fever, cough, shortness of breath or chest discomfort. He denied having night sweats or weight loss. He was not a smoker and did not consume alcohol. Initial physical examination revealed a tender swollen right lower limb extending from the midthigh to the lower leg. Vital signs and system examination were unremarkable. His initial blood investigation was normal except for a random blood glucose level of 13.7 mmol/L and elevated C-reactive protein (CRP) of 88.1 mg/L (normal range 0-5; Table 1). Doppler ultrasound of the affected limb demonstrated extensive DVT involving the right femoral vein and tibial vein (Figures 1 and 2).

**Table 1:** Initial laboratory values HI: high.

Group	Detail	Value w/units	Flags	Normal range
General hematology	White blood cells	6.0 × 10^3^/µL		4.0-10.0
	Red blood cells	6.0 × 10^6^/µL	HI	4.5-5.5
	Hemoglobin	18.3 g/dL	HI	13.0-17.0
	Platelet	147 × 10^3^/µL	LOW	150-400
	Absolute neutrophil count	4.3 × 10^3^/µL		2.0-7.0
	Lymphocyte	0.8 × 10^3^/µL	LOW	1.0-3.0
	Monocyte	0.9 × 10^3^/µL		0.2-1.0
	Eosinophil	0.0 × 10^3^/µL		0.0-0.5
	Basophil	0.04 × 10^3^/µL		0.02-0.10
Blood chemistry	Procalcitonin	0.13 ng/mL	NA	
	Bilirubin total	28 µmol/L	HI	0-21
	Total protein	80 g/L		66-87
	Albumin	32 g/L	LOW	35-52
	Alkaline phosphate	65 U/L		40-129
	Alanine aminotransferase	16 U/L		0-41
	Aspartate aminotransferase	19 U/L		0-40
	HbA1C%	8.7%	NA	
	Urea	2.8 mmol/L		2.8-8.1
	Creatinine	71 µmol/L		62-106
	Glucose random	13.7 mmol/L	HI	5.4
	C-reactive protein	88.1 mg/L	HI	0.0-5.0
	Lactic acid	1.6 mmol/L		0.5-2.2

**Figure 3:**
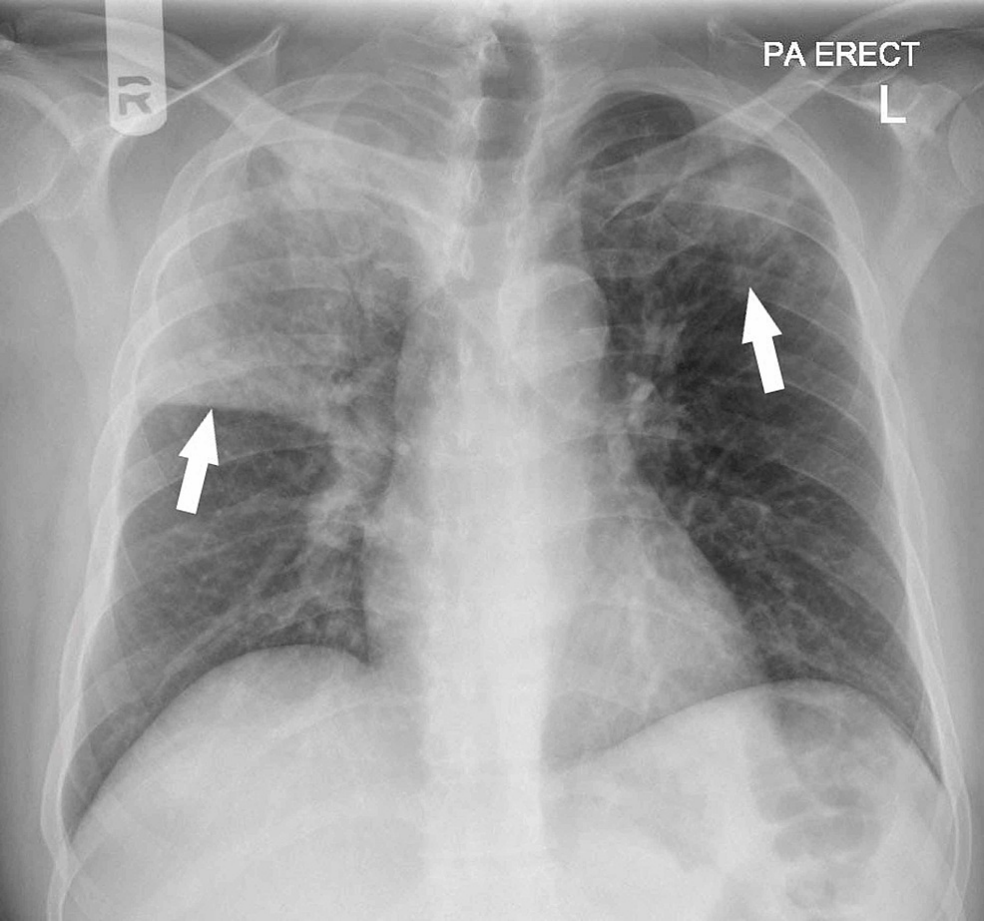
Chest X-ray demonstrating bilateral upper lobe consolidation (white arrows), more extensive on the right

**Figure 4:**
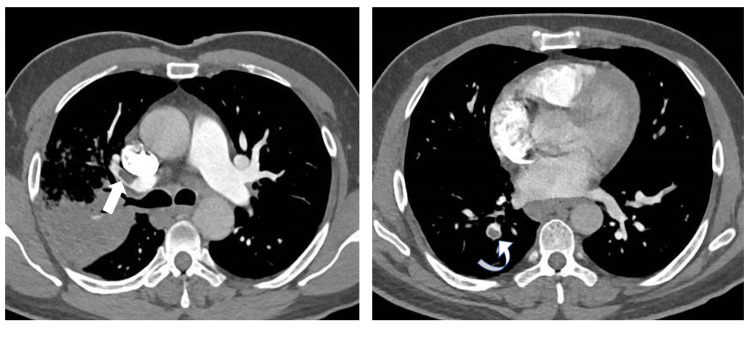
CT pulmonary angiogram demonstrates a filling defect in the anterior segmental branch of the right upper lobe pulmonary artery (straight arrow) and posterior basal segmental branch of the right lobe pulmonary artery (curved arrow)

As a part of the evaluation of unprovoked DVT, a chest X-ray was done which showed a non-homogenous opacity of the middle and upper zones of the right lung (Figure 3). The patient underwent a computer tomography (CT) scan of the chest, which revealed PE involving the bifurcation of the right pulmonary artery and segmental branches of right lower lobe arteries and bilateral upper lobe consolidation (Figure 4). In view of newly diagnosed diabetes and upper lobe consolidation, sputum acid-fast bacillus (AFB) was requested which turned out to be positive, subsequently, antituberculous treatment (ATT) was started. For thromboembolism, he was initiated on enoxaparin and later switched to warfarin. The warfarin dosage was titrated to attain a therapeutic range of International Normalized Ratio (INR; between 2 and 3). A higher dose of warfarin (18 mg) was required to attain the target INR.

**Figure 5:**
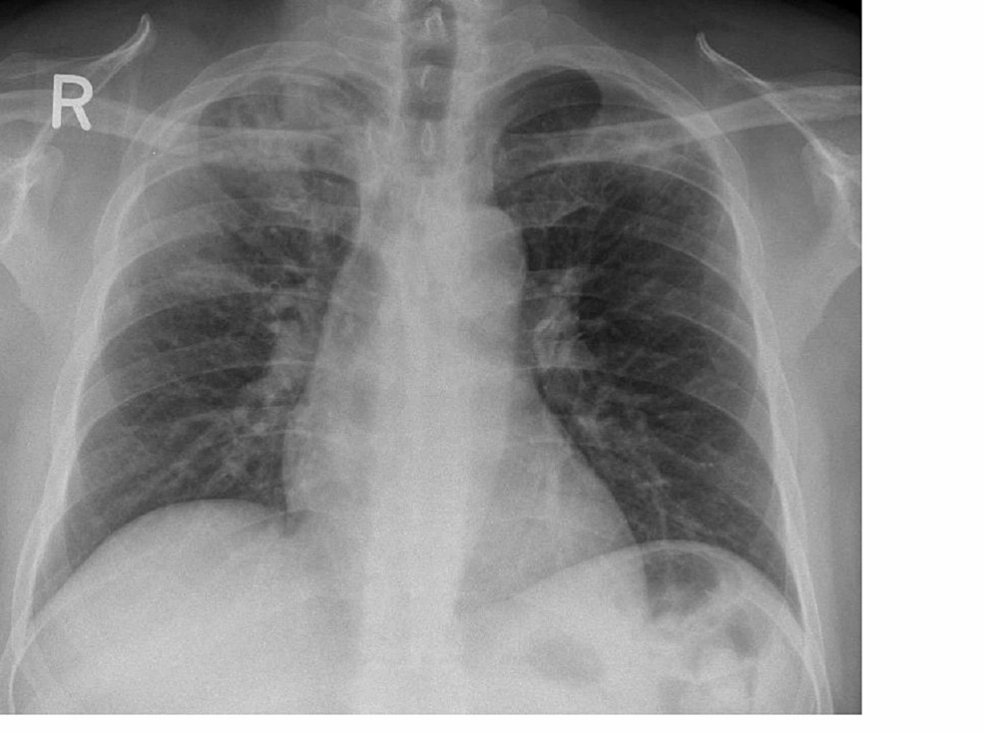
Chest X-ray on follow-up shows improvement of the consolidation

The patient was evaluated for thrombophilia and autoimmune diseases, which were negative (Table 2). He responded well to the first line of ATT medications and the repeat sputum AFB smear was negative after two weeks. He was discharged after 24 days of hospitalization. The patient was followed up in the outpatient clinic after 10 weeks. He was asymptomatic. Repeat chest X-ray during follow-up showed resolution of the consolidation (Figure 5).

**Table 2:** Other investigations PCR: polymerase chain reaction, ANA: antinuclear antibodies, CTD: connective tissue diseases, LC: liquid chromatography with tandem mass spectrometry, MTB: *Mycobacterium tuberculosis, *GPL: immunoglobulin G [IgG] phospholipid units, MPL: immunoglobulin M [IgM] phospholipid units, ABN: abnormal, CRIT: critical.

Group	Detail	Value w/units	Flags	Normal range
Autoimmune diseases	Anticardiolipin Ab IgG	5.20 GPL	NA	
Autoimmune diseases	Anticardiolipin Ab IgG Int	Negative	NA	
Autoimmune diseases	Anticardiolipin Ab IgM	5.70 MPL	NA	
Autoimmune diseases	Anticardiolipin Ab IgM Int	Negative	NA	
Autoimmune diseases	ANA CTD Int	Negative	NA	
General immunology	C3	1.57 gm/L		0.90-1.80
General immunology	C4	0.50 gm/L	HI	0.10-0.40
Homocysteine plasma	Homocysteine plasma LC-MSMS	19.9 µmol/L	HI	0.0-15.0
Mycobacteriology	Acid-fast bacilli smear	Review result	Review result	
Mycobacteriology	Acid-fast bacilli culture	Review result	Review result	
Acid-fast bacilli PCR	TB PCR	Positive	ABN	
Acid-fast bacilli PCR	MTB	MTB DNA detected	CRIT	
Acid-fast bacilli PCR	Rifampicin*-*resistance	Rifampicin resistance not detected	NA	

## Discussion

Tuberculosis remains a major public health problem, especially, in developing countries. A wide spectrum of disease presentation and multisystem involvement may sometimes cause a diagnostic dilemma for the treating physician. Thrombogenic potential and vascular complications of tuberculosis are often overlooked when evaluating patients with thromboembolism [1]. The occurrence of PE in patients with tuberculosis was described in the literature as early as 1950. Moran studied autopsy reports of patients with active tuberculosis and reported that 24.3% had PE [4]. In a report from Vaideeswar and Deshpande, on evaluation of 30 patients with aortic thrombosis, six patients had active tuberculosis [5]. The pathogenesis of thromboembolism in patients with TB can be multifactorial. All three elements of Virchow’s triad are activated in patients with tuberculosis. The hypercoagulable state is due to the increased levels of fibrin and platelet hyperactivity. Dysfunction of fibrinolysis also contributes to a hypercoagulable state. Stasis of the blood occurs due to external compression of the blood vessels by lymph nodes or from prolonged immobilization. The third factor of Virchow’s triad is the damage to the vascular endothelium due to the ongoing systemic inflammatory processes [6-8].

The time of occurrence of venous thromboembolism can be varied in patients with TB. Robson et al. reported that only 2 of the 35 patients had DVT on admission, similar to our patient and all the rest developed DVT after one week [7]. In another study, Ambrosetti et al. reports the prevalence of DVT in patients with TB as 0.6% in the initial month of treatment [9]. According to the literature, the occurrence of DVT and PE in patients with TB is uncommon, and the likelihood of both being present in the same patient is even rarer. In a case series by Mohan et al., out of five patients with either DVT or PE in pulmonary tuberculosis, only one had both the entities [3].

During the management of venous thromboembolism in patients with tuberculosis, the enzyme-inducing property of rifampicin which is used as the first line of therapy causes a challenge in attaining therapeutic anticoagulation while using warfarin [10]. Patients often require a higher dose of warfarin as in our patient and prolonged duration of heparin till a therapeutic level of INR is attained.

## Conclusions

Tuberculosis is a common infectious disease in the developing world and can manifest with various pulmonary or extrapulmonary symptoms. Tuberculosis can be considered as an independent risk factor for the development of venous thromboembolism. The etiology for the development of thromboembolism is multifactorial. Early diagnosis and treatment of this serious complication are crucial for a better outcome.
